# A Mechanism of DC-AC Conversion in the Organic Thyristor

**DOI:** 10.3390/ma3032027

**Published:** 2010-03-19

**Authors:** Tomohiro Suko, Ichiro Terasaki, Hatsumi Mori, Takehiko Mori

**Affiliations:** 1 Department of Applied Physics, Waseda University, 3-4-1 Ohkubo, Shinjuku-ku, Tokyo 169-8555, Japan; E-Mail: tomohiro0120@toki.waseda.jp (T.S.); 2 The Institute for Solid State Physics, The University of Tokyo, 5-1-5, Kashiwanoha, Kashiwa 277-8581, Japan; E-Mail: hmori@issp.u-tokyo.ac.jp (H.M.); 3 Department of Organic and Polymeric Materials, Tokyo Institute of Technology, 2-12-1, Ookayama, Meguro-ku, Tokyo 152-8552, Japan; E-Mail: mori.t.ae@m.titech.ac.jp (T.M.)

**Keywords:** organic conductor, charge order, nonlinear conduction, current oscillation

## Abstract

The charge ordered organic salt *θ*-(BEDT-TTF)_2_CsZn(SCN)_4_ exhibits a giant nonlinear conduction at low temperatures. The voltage-current characteristics of this compound are similar to those of a thyristor device, after which we named it the organic thyristor. This material shows current oscillation in the presense of dc voltage, which arises from a mechanism different from conventional oscillating circuits, because the oscillation appears in a sample that does not show negative derivative resistance. We have performed a standard circuit analysis, and show that the voltage-current curve is “blurred” in the high current region, and the oscillation occurs in the blurred region. This type of oscillation has never been reported, and a possible origin for this is suggested.

## 1. Introduction

The organic electronic materials have attracted a keen interest because they show various features that inorganic materials do not share. For example, the organic materials are mainly composed of C, H and O, being free from rare metals. Some years ago, we found giant nonlinear conduction in the organic salt *θ*-(BEDT-TTF)_2_Cs*M’* (SCN)_4_ (*M’* = Co and Zn) at 4 K [1,2], which was later verified by other groups [3,4]. Since the voltage-current characteristics were similar to those of a thyristor device, we named this material the organic thyristor [2]. In addition to the giant nonlinear conduction, we found that this material showed a 40 Hz current oscillation in the presence of dc bias [2]. This dc-ac conversion (inverter effect) indicates that the organic thyristor can be used for an oscillating circuit like a conventional thyristor device. After this discovery, the nonlinear conduction in organic conductors has received a renewed attention [5,6,7], although many of organic materials were recognized for non-ohmic conductors long before. [8,9,10,11,12,13] The current oscillation has been also searched and discovered in various strongly correlated systems [14,15].

The organic salt *θ*-(BEDT-TTF)_2_*M M’*(SCN)_4_ (*M* = Cs, Rb; *M’* = Co, Zn) is a layered material known as a charge-ordered conductor [16], which has been extensively investigated for the last decade [17,18]. The charge ordered state was first investigated in the related salt *θ−*(BEDT-TTF)_2_RbZn(SCN)_4_. In this material, the charge ordering specified by a wave number of (0, 0, 1/2) takes place below a transition temperature of 195 K, which is verified by magnetic resonance [19], x-ray diffraction [20], infrared spectroscopy [21] and Raman spectroscopy [22]. In this charge ordered state, holes are localized at every two molecules along the *c* axis, and the energy gap is opened at the Fermi level. In contrast, the charge ordered state is not well defined in the organic thyristor. Watanabe *et al.* [23] found that the two kinds of charge ordered domains specified by wave numbers of (0, 0, 1/2) and (2/3, 0, 1/3) coexist down to low temperatures. This state is similar to a cluster glass of disordered ferromagnets, where all the holes are frozen in nano-size domains of one of the two charge-ordered phases. We think that this intrinsically inhomogeneous state [24] has something to do with the nonlinear conduction of this material, but the nature of this state is still controversial [25,26,27,28].

At present, the mechanism of the 40-Hz current oscillation is totally unknown. An important point is that this is different from the narrow band noise observed in the sliding motion of the charge density wave, because the oscillation frequency is independent of dc bias [29,30,31,32]. We should also note that a well-defined thermodynamic phase transition is not observed in the organic thyristor [33], which suggests that the phase of the charge order does not have long range order. In such situations, the order parameter does not acquire the rigidity, and concomitantly does not show sliding motion either [34]. In this article we have performed a standard circuit analysis to the ac component of the current oscillation, and suggest a possible mechanism of the dc-ac conversion in the organic thyristor.

## 2. Experimental

Thin plate-like single crystals of *θ−*(BEDT-TTF)_2_CsZn(SCN)_4_ were prepared by a galvanostatic anodic oxidation method described in [16]. In this article, we compare two samples. The first one is called Sample B1 which shows negative derivative resistance like a thyristor device. The second sample is called Sample B2 which shows nonlinear resistance, but does not show negative derivative resistance. The letter “B” represents the measurement direction, *i.e.*, the external current *I*_ext_ and the external voltage *V*_ext_ were applied along the *b* direction that is perpendicular to the conducting BEDT-TTF layer. Considering that the nonlinear conduction is similarly observed along the *c*- and *b*-axis directions [35], we focused the *b*-axis direction because of the higher resistivity. Since the current oscillation experiment requires a two-probe configuration in constant voltage conditions, the sample resistance should be sufficiently higher than the contact resistance. The contact resistance of the samples was carefully evaluated to be about 10–50 Ω, which was 100-times lower than the sample resistance at 4.3 K and was able to be safely neglected.

The dimensions of Sample B1 were 0.47 *×* 0.09 *×* 1.45 mm^3^ along the *a*, *b*, and *c* axes, while those of Sample B2 were 0.38 mm^2^ (*ac* plane) *×* 0.05 mm (*b* axis). Gold wires of 20 *µ*m diameter were attached with gold paste to Sample B1, and with carbon paste to Sample B2 on the ac surfaces. Although one can easily convert the resistance, current and voltage into resistivity, current density and electric field by using these dimensions, we dare to show only raw values for the sake of clear understanding.

The voltage-current characteristics and the current oscillation were measured in a two-probe configuration in series with a standard resistance (*R*_std_) of 0.5–10 kΩ in a liquid He cryostat. All the measurements were done at 4.3 K, because the oscillation disappeared when the sample was dipped in liquid He. The oscillation was observed in a narrow range of temperature, from 4.3 to 4.8 K, whose frequency increased with increasing temperature (38–40 Hz at 4.3 K and 50–55 Hz at 4.8 K). In the constant current measurement, the external current *I*_ext_ was applied using a current source (Keithley 6221) and the sample voltage *V*_sample_ was measured with a nanovolt meter (Keithley 2182). In the constant voltage measurement, the constant voltage *V*_ext_ was applied using a function generator (nF WF1965) across the sample and the standard resistor *R*_std_ connected in series, and the sample current *I*_sample_ was measured by a voltage drop across the standard resistor *R*_std_*I*_sample_, where the sample voltage was obtained by *V*_sample_ = *V*_ext_*−* *R*_std_*I*_sample_. The current oscillation was recorded by using a digital oscilloscope (Tektronics TDS3012B) through the voltage drop across the standard resistor.

## 3. Results and Discussion

**Figure 1:**
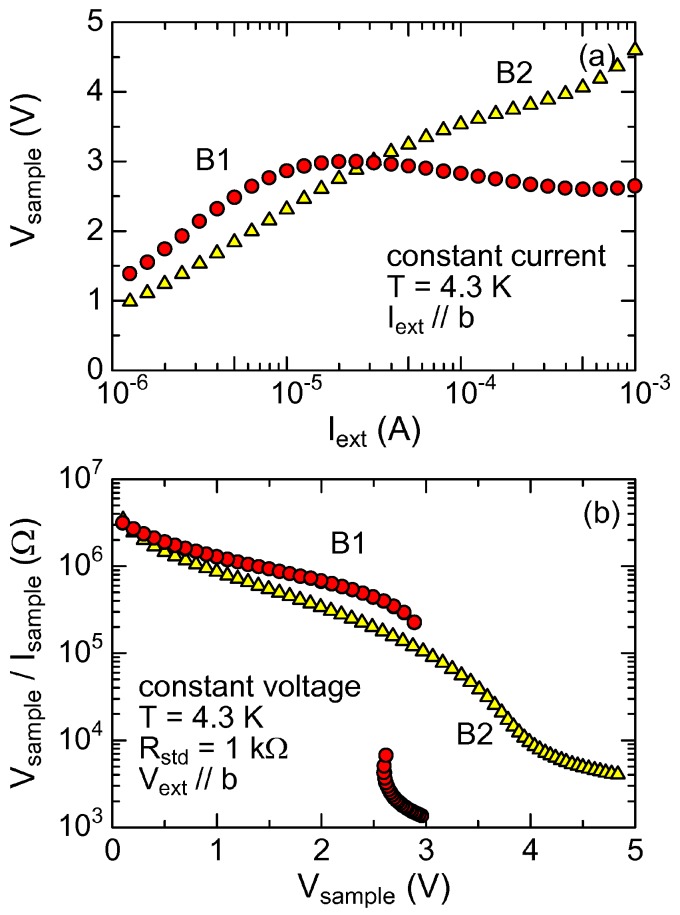
(a) Voltage-current characteristics of Samples B1 and B2 measured in constant currents at 4.3 K. (b) Resistance *V*_sample_*/I*_sample_ of Sample B1 and B2 plotted as a function of the sample voltages *V*_sample_ measured in constant voltage at 4.3 K.

Figure 1(a) shows the voltage-current characteristics of the two samples at 4.3 K in constant currents. Sample B1 shows negative derivative resistance (*dV*_sample_*/dI*_ext_*<* 0) from *I*_ext_ = 10 to 500 *µ*A, which is essentially similar to those reported by Sawano *et al.* [2]. In contrast, the voltage of Sample B2 increases with increasing current, and shows a slight kink around 200 *µ*A, although the response of Sample B2 is highly nonlinear. We have no clear idea of a cause for the different characteristics, but single crystal samples have uncontrollable imperfections, which may affect the voltage-current characteristics. A similar case is seen in single crystal samples of the charge-density-wave oxide K_0.3_MoO_3_; Some samples show switching behavior, and others do not [32].

The nonlinear conduction is more clearly seen in the form of resistance. Figure 1(b) shows the resistance *V*_sample_*/I*_sample_ of the two samples in the constant voltage conditions. As for Sample B1, a drastic jump is seen around 3.0 V, which is similar to the nonlinear resistance reported by Sawano *et al.* [2]. In the present measurement, the resistance was recorded only in increasing voltage, so that no hysteresis is seen in Figure 1(b). The abrupt jump indicates that the sample resistance is a multi-valued function of voltage. The resistance of Sample B2 is also nonlinear, but smoothly decreases with increasing external voltage with a broad step around 3.5 V.

Here we briefly comment on the effect of Joule heating. In Sample B2, the nonlinear resistance is observed below 1 V, which corresponds to a Joule heat less than 2–3 *µ*W. Previously we examined a possible temperature increase by using a standard heat transfer theory, and evaluated it to be 0.2 K for 0.1 mW at maximum [35]. According to this evaluation, the nonlinear conduction in Sample B2 below 1 V seems difficult to be explained by a temperature increase of 0.5 mK. The discussion on the high current region is indeed subtle, but the voltage-current curve observed experimentally is not simply due to self-heating (see Figure 4(b) and the corresponding discussion).

**Figure 2:**
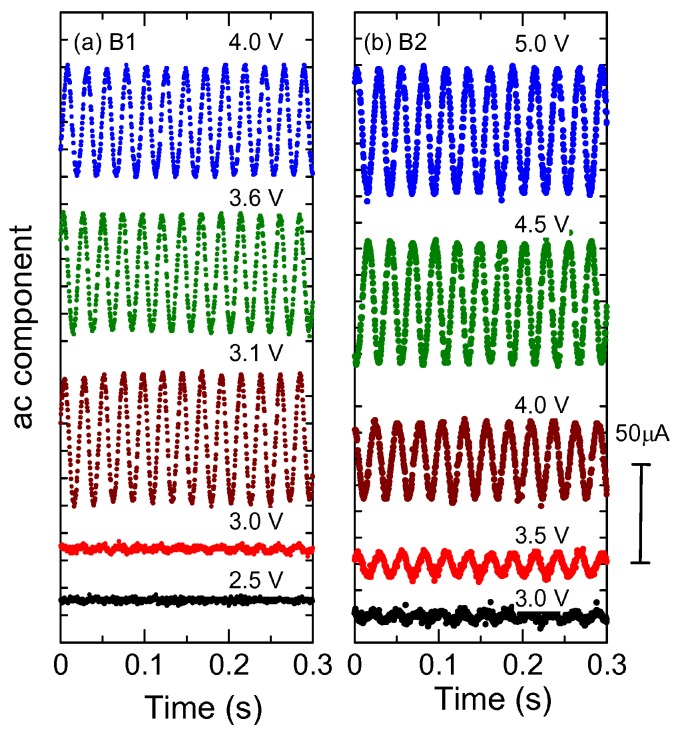
The current oscillation in various constant voltages (*V*_ext_) for (a) Sample B1 and (b) Sample B2. The standard resistance is 1 kΩ, and the temperature is 4.3 K. Sample B2 does not show the negative derivative resistance in the voltage-current characteristics (Figure 1(a)), but show clear current oscillations.

Figure 2(a) shows the current oscillation of Sample B1 at various external voltages for *R*_std_ = 1 kΩ at 4.3 K. The ac component of the sample current shows a small ripple as a precursor of the 40-Hz oscillation below 3 V, and a clear ac current with a large amplitude of 20–25 *µ*A suddenly appears above 3.1 V. The frequency is around 40 Hz, and is independent of dc bias. This is different from the sliding motion in the charge density waves [29,30,31,32], and is also different from the current oscillation in other materials [14,36]. We inserted various capacitances connected in parallel to the sample, but the oscillation frequency did not change with external capacitance (the data are not shown). This is also different from the narrow band noise in the charge density wave [32] or the current oscillation in the Mott insulator VO_2_ [37].

Most unexpectedly, similar current oscillation is seen in Sample B2 that has no negative derivative resistance. As shown in Figure 2(b), the current oscillation continuously grows with increasing external voltage. This is seriously incompatible with our previous explanation that the mechanism of the current oscillation is similar to an inverter circuit, in which a positive feedback loop is made using bistable states of a switching device and a phase retardation of a capacitance. Kishida *et al.* [38] have created current oscillation making an oscillation circuit from a capacitance and an organic conductor showing negative derivative resistance. The oscillation in VO2 is similarly explained by Kim *et al.* [37]. In this scenario, the bistability due to negative derivative resistance is essential to the oscillation, which cannot explain the results in Figure 2(b).

In order to understand the dc-ac conversion effect at least in a phenomenological level, we analyze the amplitude of the oscillation quantitatively. Since the oscillation is almost sinusoidal, the sample current can be expressed by

**Figure 3:**
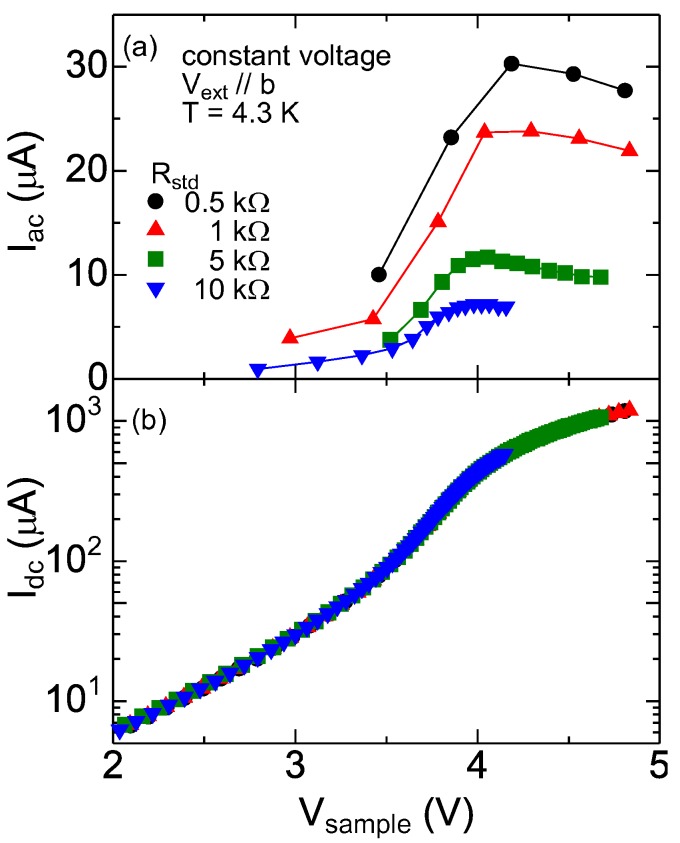
(a) The ac component *I*_ac_ and (b) dc component *I*_dc_ of the current oscillation in Sample B2 plotted as a function of sample voltage *V*_sample_ measured in constant voltages. Note that the ac component strongly depends on standard resistance *R*_std_.

*I*_sample_ = *I*_dc_ + *I*_ac_ sin *ωt*

where *I*_ac_ and *I*_dc_ are the ac and dc components, and *ω* is the angular frequency (*∼*80*π* Hz). ¿From the measured oscillation, one can obtain the maximum (*I*_max_) and minimum (*I*_min_) of the sample current can determine *I*_dc_ and *I*_ac_. Figure 3(a) and Figure 3 (b) show *I*_ac_ = (*I*_max_*−* *I*_min_)*/*2 and *I*_dc_ = (*I*_max_ + *I*_min_)*/*2 in Sample B2 plotted as a function of sample voltage, respectively. As shown in Figure 3(b), *I*_dc_ is independent of *R*_std_. This is reasonable, because the voltage-current characteristics must be unaffected by *R*_std_. In contrast, the results seen in Figure 3(a) are highly nontrivial. *I*_ac_ strongly depends on the value of the standard resistance. One can find that the maximum of *I*_ac_ for *R*_std_ =0.5 kΩ is almost one order of magnitude larger than that for *R*_std_ = 10 kΩ.

By using the data in Figure 3, let us specify the operating points of the organic thyristor on the *I*_sample_*−* *V*_sample_ plane using a standard circuit analysis. ¿From Kirchhoff’s laws, *V*_sample_ and *I*_sample_ satisfy

**Figure 4:**
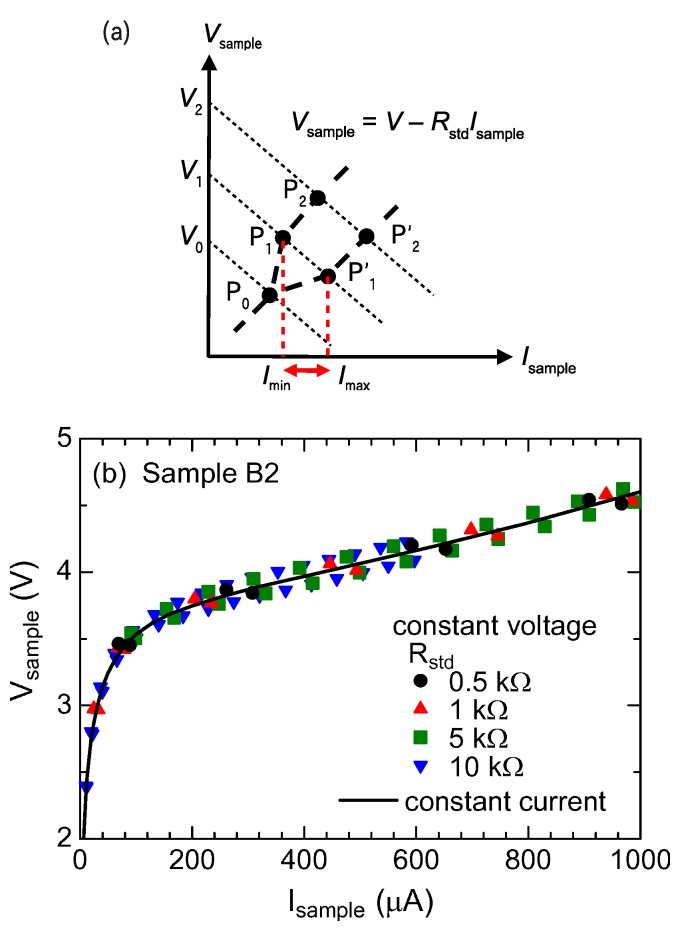
(a) Schematic figure of the operating points in the current-voltage plane (see text). (b) Operating points determined from the experimental data of Sample B2. The solid curve represents the voltage-current characteristics measured in constant currents.

*V*_sample_ = *V*_ext_*−R*_std_*I*_sample_ (1)

and are expressed by a point on the line with an intercept of *V*_ext_ and a slope of *−R*_std_. As is schematically drawn in Figure 4(a), we sweep the *I*_sample_*−* *V*_sample_ plane with parallel lines by changing *V*_ext_ with a fixed value of *R*_std_. When *V*_ext_ is smaller than a threshold voltage *V_c_*, the current oscillation does not take place. In this case, one stable solution (the only one operating point) is specified by the intersection point of the line and the nonlinear voltage curve *V*_sample_(*I*_sample_) (P_0_ in Figure 4(a)). When *V*_ext_ exceeds *V_c_*, we can plot two operating points in the plane by measuring *I*_max_ and *I*_min_. By sweeping *V*_ext_, we can successively determine two operating points as are indicated by P*_i_* and P’*_i_* (*i*=1, 2, *· · ·*) in Figure 4(a). Consequently we can draw the two voltage curves by connecting the operating points as P_0_*−*P_1_*−*P_2_*−*⋯ and P_0_*−*P’_1_*−*P’_2_*−*⋯. In other words, the voltage-current characteristics oscillates between the two curves.

Figure 4(b) shows the operating points thus obtained in the *I*_sample_*−* *V*_sample_ plane. In spite of various conditions (*R*_std_ =0.5-10 kΩ and *V*_ext_ =0-10 V), all the plots are consistently distributed, as if the voltage curve were blurred above 100 *µ*A. Such voltage-current characteristics have never been reported before, and strongly indicate a unique property of the current oscillation of this material.

We notice several features from Figure 4(b). First, the voltage curve is not due to self-heating. According to the previous work [35], the resistance depends on temperature in the low current region rather than in the high current region. Or equivalently, the voltage depends on temperature for low currents rather than for high currents. If the current oscillation were simply due to self heating, the voltage-current curve would be expressed by a combination of curves at different temperatures. In this case, the blurred curve should be seen in the low current region, which is the opposite situation to Figure 4(b). Second, the blurred curve well explains the *R*_std_ dependence of *I*_ac_ in Figure 3(a). Since the blurred curve has a roughly constant width in voltage (*∼*0.1–0.2 V), the difference of the working voltage points *V*_max_*−* *V*_min_ is roughly constant. This means that *I*_max_*−* *I*_min_ = (*V*_max_*−* *V*_min_)*/R*_std_ is roughly inversely proportional to *R*_std_. Third, the nonlinear resistance is not simply due to the melting of the charge ordered domains, because the operating points are so close that the “volume fraction” of the melted domains does not change much. Very recently, Ajisaka *et al*. [39] have proposed a theory of nonequilibrium Peierls transition, which can explain the nonlinear conduction of the organic thyristor semi-quantitatively. Within this framework, the suppression of the X-ray diffuse scattering does not always mean the melting of the charge order, but suggests the reduction of the charge-order gap [40]. In this respect, the current oscillation may be caused by the amplitude modulation of the charge order gap in space and time.

Finally we make a brief comment on an origin of the blurred curve. Watanabe *et al.* [20] have analyzed the crystal structure of *θ−*(BEDT-TTF)_2_RbCo(SCN)_4_ below and above the charge ordering temperature *T_c_*, and have found that the BEDT-TTF molecules rotate below *T_c_*. Thus we expect that the current suppresses the rotation, when the current suppresses the charge order. Although there is no well-defined *T_c_* in the organic thyristor *θ−*(BEDT-TTF)_2_CsZn(SCN)_4_, we expect that there exists a similar electron-lattice interaction, and that the electrical current can change the electric field and/or the current density through the distortion of the unit cell. If so, the voltage curve will be slightly changed in the high current region. We should note that quartz oscillators exhibit a MHz voltage oscillation through the lattice distortion by electric field (the Piezoelectric effect in this case). The Debye temperature of the organic thyristor is much lower than that of quartz, and thus a low frequency of 40 Hz is not surprising. To examine this idea, the X-ray diffraction measurement in electric field is essential, which is now in progress.

## 4. Summary

We have observed the current oscillation in the two samples of *θ*-(BEDT-TTF)_2_CsZn(SCN)_4_, one of which showed the oscillation without negative derivative resistance. This clearly suggests that the current oscillation is caused by a mechanism different from conventional oscillating circuits. Based on a conventional electrical-circuit analysis, we have obtained the voltage-current curve experimentally, when the 40-Hz oscillation takes place. The voltage-current curve is found to have a finite width in the high current region, which has never been reported before. We propose that the oscillation is due to the current-induced lattice distortion, which is essentially the same as the mechanism of quartz oscillators.
